# Is clinical experience important for obtaining the primary stability of dental implants with aggressive threads? An ex vivo study

**DOI:** 10.4317/medoral.22733

**Published:** 2019-03

**Authors:** Onur Geckili, Hakan Bilhan, Esma Geckili, Ece Barca-Dayan, Cagatay Dayan, Canan Bural

**Affiliations:** 1 PhD, DDS, Professor, Department of Prosthodontics, Istanbul University, Faculty of Dentistry, Istanbul, Turkey; 2 PhD, DDS, Department of Periodontology, Faculty of Health, School of Dentistry, Universitat Witten/Herdecke, Witten, Nordrhein-Westfalen, Germany; 3 PhD, DDS, Istanbul Bilgi University, Department of Dentistry Services, Oral and Dental Health, Istanbul, Turkey; 4 DDS, PhD Student, Department of Periodontology, Istanbul University, Faculty of Dentistry, Istanbul, Turkey; 5 PhD, DDS, Program of Dental Technicians, Istanbul University Cerrahpasa, Istanbul, Turkey

## Abstract

**Background:**

The aim of this study was to investigate the clinicians’ experience on maintaining the primary stability of implants with aggressive threads belonging to a novel dental implant system.

**Material and Methods:**

Three hundred implants with aggressive threads were inserted in fresh bovine ribs mimicking Type IV bone by five clinicians which were classified according to their previous experience of total number of implant insertion. An independent examiner measured the primary stability of all implants after insertion by using resonance frequency analysis (RFA), electronic percussive testing (EPT) and removal torque methods.

**Results:**

No significant differences were detected between the stability values measured by the clinicians (*p*< 0.05) except the Periotest values (PTVs) of the non-experienced clinician. PTVs of the non-experienced clinician were significantly higher than the PTVs of the expert and good clinicians (*p* >0.05). Significantly higher stability values were detected in the secondary insertion of the non-experienced clinician as compared to her initial insertion values (*p* >0.05). No significant differences were detected between the first and second measurements of the other clinicians (*p*< 0.05).

**Conclusions:**

Within the limitations of this ex-vivo study, it may be concluded that experience does not play an important role in maintaining the stability of implants with aggressive threads.

** Key words:**Implantology, experimental design, osseointegration.

## Introduction

Treatment of any kind of edentulism with dental implants have emerged to be a predictable option in dentistry. For achieving a successful implant treatment, dental implants should integrate to the surrounding bone which has been termed as osseointegration (1). To accomplish osseointegration, it has been well documented in the literature that implants should be adequately stabile in the bone both after the surgery and during the healing period (2-5). Implant stability has been recently defined by Trisi and collegues (6) as “the value of relative micromotion between the implant and the surrounding bone.” Avoiding this micromotion and maintaining the stability immediately after surgery which is so-called as initial or primary stability depends on several factors such as the quality and quantity of the surrounding bone, the surgical technique used and the implant design (7-9).

Measuring the stability after the surgery (secondary stability) is very important to decide the time of loading of an implant. Additionally, unusual changes in the stability of an implant during the healing period or after osseointegration ought to alert the clinicians to take some precautions such as unloading the implant, avoiding a possible occlusal trauma or infection control (4,10).

Several methods have been proposed to measure implant stability. Of these, only resonance frequency analyses (RFA) and electronic percussive testing (EPT) are regarded as clinically applicable both to measure the primary stability and to monitor the changes in the stability during the healing period (11,12). Insertion torque method is also a safe one but can only be used to evaluate the primary stability of an implant (12). Reverse torque method (RT) is not clinically applicable because it may negatively affect the stability and loosen the implant; but gives valuable numerical information in experimental tests (13,14).

It has been shown that the clinical performance of health technologies improves over time, as the clinicians become more familiar with the presented technology which is also termed as the learning curve (15,16).

The skill and experience of the clinicians is of high importance in providing a successful implant treatment. It was shown that clinician’s inexperience makes the treatment complex and only experienced clinicians should achieve harder protocols like immediate loading (15). In a previous retrospective research performed by the present authors it was shown that due to the improvement of the skill of the surgeon, implants that had been installed 5 years ago or earlier had a higher failure rate than those that were inserted more recently (17). However, Jemt *et al.* (18) couldn’t find any differences in failure rates of inexperienced surgeons placing as compared with experienced surgeons in a more recent research covering larger number of patients.

The factors that may influence the primary stability of dental implants have been investigated in many clinical and in vitro studies (4,5,9,10). Implant design, surgical technique modifications and host bone quality and density have been reported to positively affect the primary stability of an implant (9). However there’s little information about the clinician’s experience on providing the primary stability. Therefore the present *ex-vivo* study was conducted to investigate this effect on a novel dental implant system.

## Material and Methods

Fresh bovine bone ribs belonging to the same animal were found from a butcher’s shop and carefully chosen for the experimental procedures. The bovine bone ribs were similar to type IV quality bone (19,20) according to the Lekholm and Zarb classification (21).

A total of 300 implants were inserted into the selected bovine bone ribs with a safe distance to each other by 5 different clinicians categorized based upon their clinical expertise on dental implant insertion as follows:

1. Expert: Clinician having an experience of more than 2000 implants

2. Qualified: Clinician having an experience of 1000-2000 implants 

3. Good: Clinician having an experience of 500-1000 implants 

4. Novice: Clinician having an experience of 100-500 implants

5. Inexperienced: Clinician that had never placed any implant

The implants were all 4.1 mm wide and 11.5 mm long and belonged to the same manufacturer (Mode Rapid Implants; Mode Medikal, Istanbul, Turkey). Since the implant system was new in the market, the implant system was introduced (the sequence of drills and implant insertion) by a short briefing prior to the experimental procedures to all involved clinicians.

First 4 clinicians prepared the implant beds following the standard drilling protocol recommended by the manufacturer and inserted the implants with their own experience. The 5th clinician performed the implant bed preparation and implant insertion under the supervision of a qualified company representative.

30 implants were inserted into the ribs by each clinician. After 24 hours, another 30 were inserted by each clinician in order to measure the learning effect on providing implant stability.

-Measurements

After the insertion of all 300 implants, an independent examiner blinded to the study protocol and calibrated before the study made all the stability measurements using the RFA, EPT and RT methods.

**Figure 1:**
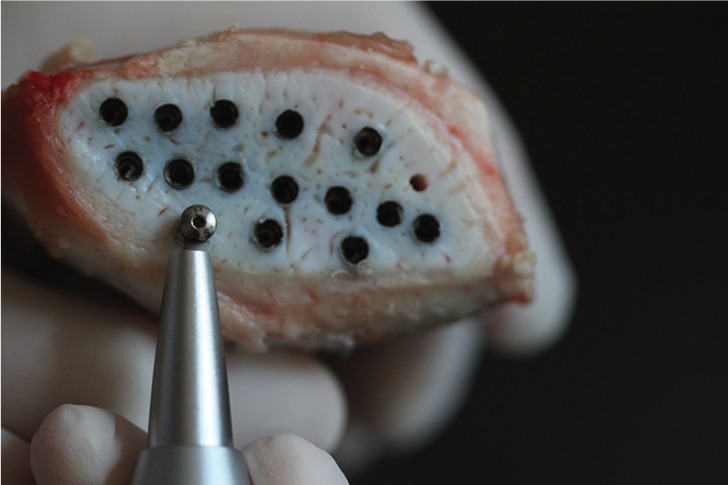
Measurement of the implant stability by using RFA method.

RFA was measured using the magnetic OsstellTM ISQ (OM2; OsstellTM ISQ, Integration Diagnostics, Savedalen, Sweden). A magnetic peg pre-calibrated for Mode implants was inserted using a plastic screwdriver provided by the manufacturer (Smartpeg type 60, Integration Diagnostics, Savedalen, Sweden) and hand tightened. Osstell probe was held 1 mm away from the Smartpeg at a 90-degree angle both in the buccal and mesial directions for each implant (Fig. 1) and the RFA value was registered as implant stability quotient (ISQ) on the digital screen of the instrument. The mean of the buccal and mesial ISQs were calculated and recorded as one ISQ value for each implant.

**Figure 2:**
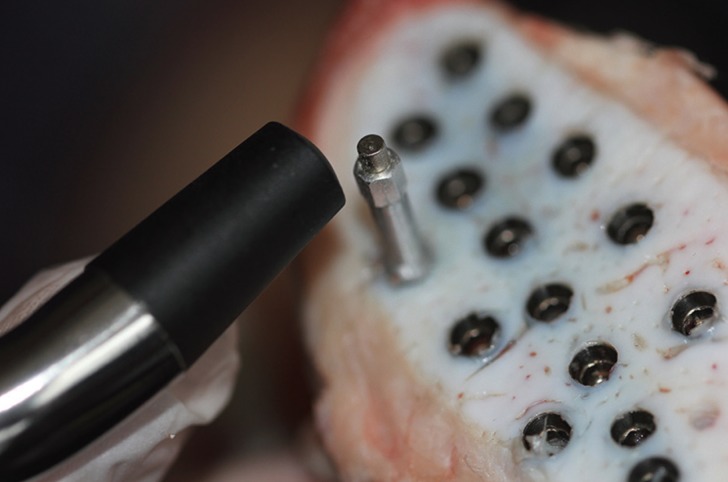
Measurement of the implant stability by using EPT method.

EPT was measured using the wireless Periotest M (PT2; Medizintechnik Gulden, Modautal, Germany). 2 mm healing abutments (Healing abutment Mode, Modemedikal) were screwed to all of the implants and the hand-piece of the device was seized perpendicular to the healing abutments in the buccal directions (Fig. 2) and after percussion the microcomputer recorded the Periotest values (PTVs).

**Figure 3:**
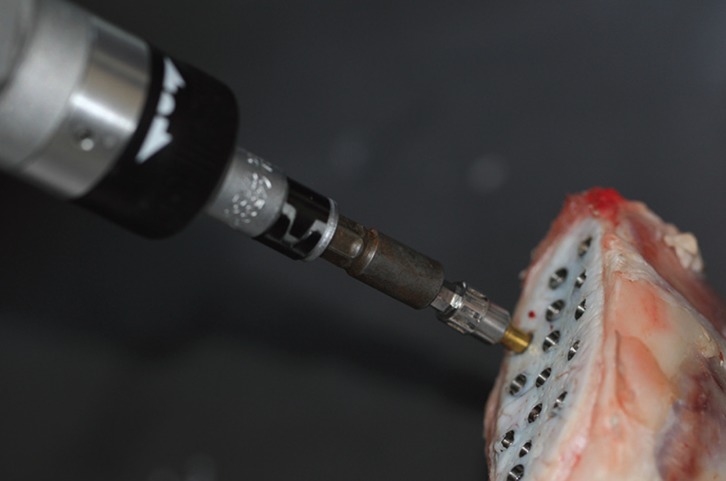
Measurement of the implant stability by using RT method.

After completing the RFA and EPT measurements, implants were loosened using a pre-calibrated digital torque gauge (Cedar DID-04, Sugisaki Meter Co.,Ltd, Japan) and one reverse torque value (RTV) was recorded for each implant (Fig. 3).

-Statistical analyses

Statistical analysis was performed using Statistical Package for Social Sciences (SPSS) for Windows software (IBM Corp. Released 2013. IBM SPSS Statistics for Windows, Version 22.0. Armonk, NY). The relevance of the parameters to the normal distribution was analyzed by using Shapiro Wilks test. One-way analysis of variance (ANOVA) was used to find significant differences between the clinicians’ stability values and the post hoc Tukey’s honestly significant difference (HSD) test and Tamhane’s T2 test were used to identify the source of the difference. The change of values between the first and second insertions was measured using Paired sample t test. Correlations between the stability values measured by the three instruments were assessed using the Pearson correlation test. The level of significance was set at 95% (*p*<0.05)

## Results

The means of the PTVs, ISQs and RTVs are presented in  Table 1, no significant differences were observed between the ISQs and RTVs of the clinicians (*P* > .05); but the PTVs of the inexperienced clinician were significantly higher (lower stability) than the expert and qualified clinicians (*P* = .008 and *P* = .001 respectively). No other significance was observed between the PTVs of other clinicians (*P* > .05).

**Table 1:**
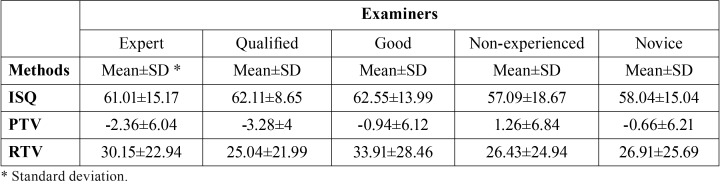
PTVs, ISQs and RTVs of the clinicians.

PTVs, ISQs and RTVs of initial and secondary insertion of the first 4 clinicians didn’t differ significantly (*P* > .05). However, significantly higher ISQs and RTVs (*P* = .001) and lower PTVs (*P* = .019) were detected in the secondary insertion of the inexperienced clinician as compared to her initial insertion values ( Table 2).

**Table 2:**
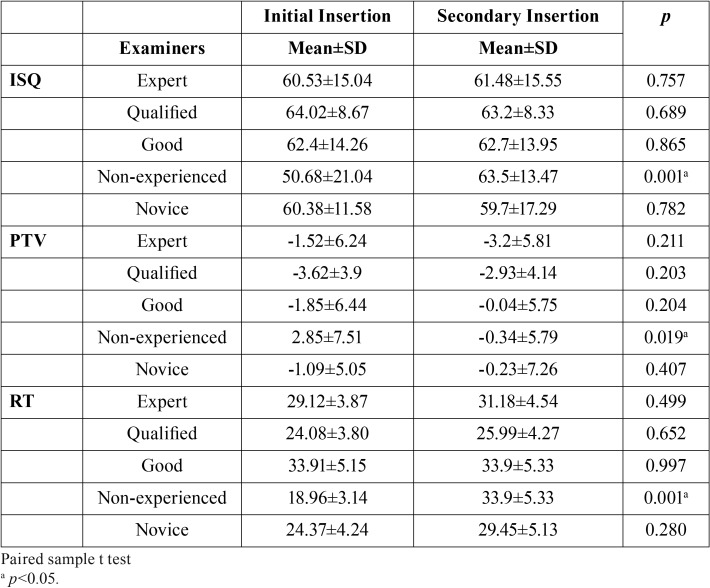
Comparison of the PTVs, ISQs and RTVs of initial and secondary insertion of the clinicians.

When all the implants were considered, Pearson correlation values showed significant positive correlations between the ISQs and RTVs (73.8%, *P* < .05) and significant negative correlations between PTVs and ISQs (83.3%, *P* < .05) and between PTVs and RTVs (59.6%, *P* < .05).

## Discussion

It is well known that primary stability is a prerequisite for obtaining successful osseointegration. Especially when the immediate or early loading protocol is intended and the implants are inserted in cancellous bone in regions such as the posterior maxilla, it is better to use implant designs with more aggressive threads in order to achieve higher stability levels. These implants have an ability to change direction during insertion that allows the clinicians to change position of the implant in order to correct the position or maintain higher stability and therefore are generally recommended for experienced users (22). The main goal of the present study was to analyze the effect of clinical experience in providing the stability of implants having the above mentioned design features. Additionally the implants used belonged to a novel dental implant system and none of the clinicians involved in this study had any experience in this new system. Therefore it was possible to measure only the effect of previous experience in implant surgery not the experience of a specific implant system.

Primary stability of an implant may be measured using several methods but the most commonly used methods are insertion torque, RFA and EPT. Since a separate investigator measured the stability of all implants in the present study, it was not possible to measure the insertion torque values of the implants inserted by different clinicians. Therefore RT was preferred instead together with RFA and EPT to measure implant stability which works identical with insertion torque but not clinically applicable (13,14). Pearson correlation values showed significant correlations between the methods used in the present study which is in accordance with previous studies (20).

Only one study exists in the dental literature dealing with the effect of clinical experience on providing dental implant stability. In this *ex-vivo* study similar to ours, Romanos *et al.* (23) categorized the clinicians into 3 as master, good and non-experienced and stability measurements were performed by means of EPT and RFA. In order to obtain more detailed results of experience effect on implant stability, the clinicians were divided into 5 as expert, qualified, good, novice and inexperienced in the present study. Romanos *et al.* (23) found that the inexperienced clinicians inserted parallel implants with higher ISQs and lower PTVs (higher stability) than the other clinicians. This finding is just the opposite of the findings of the present study in which the PTVs of the inexperienced clinician were higher (lower stability) than the expert and qualified clinicians. This may be due to the different implant designs used in the two studies. Implant designs with more aggressive threads which were used in the present study and generally recommended for experienced users (22) may be the reason of lower stability values of the inexperienced clinician.

In the present study, a novel dental implant system was used, with which the clinicians involved in the present study had never worked before. All the clinicians have made 2 insertions in 24 hour intervals in order to measure the effect of their experience on learning to provide better implant stability. However only the inexperienced clinician who had never inserted a single implant before the present study presented higher stability values in her second insertion. Other clinicians showed similar stability values in their first and second insertions.

Our results showed that even a novice clinician having an experience of 100-500 implants may provide implant stability as high as an expert clinician having an experience of more than 2000 implants when an aggressive threaded implant is used.

## Conclusions

Within the limitations of this study, it may be concluded that the implant geometry is more important than the experience of the clinician in order to achieve good primary stability in cancellous bone. Primary stability can be achieved even if the clinician has a minor experience in implant surgery.
